# Is Virtual Reality Training More Effective Than Traditional Physical Training on Balance and Functional Mobility in Healthy Older Adults? A Systematic Review and Meta-Analysis

**DOI:** 10.3389/fnhum.2022.843481

**Published:** 2022-03-23

**Authors:** Meng Liu, Kaixiang Zhou, Yan Chen, Limingfei Zhou, Dapeng Bao, Junhong Zhou

**Affiliations:** ^1^Sports Coaching College, Beijing Sport University, Beijing, China; ^2^College of Sports, Chengdu University of Traditional Chinese Medicine, Chengdu, China; ^3^School of Strength and Conditioning Training, Beijing Sport University, Beijing, China; ^4^China Institute of Sport and Health Science, Beijing Sport University, Beijing, China; ^5^Hebrew SeniorLife, Hinda and Arthur Marcus Institute for Aging Research, Harvard Medical School, Boston, MA, United States

**Keywords:** virtual reality training, functional mobility, balance, older adults, systematic review and meta-analysis

## Abstract

**Objective:**

The studies showed the benefits of virtual reality training (VRT) for functional mobility and balance in older adults. However, a large variance in the study design and results is presented. We, thus, completed a systematic review and meta-analysis to quantitatively examine the effects of VRT on functional mobility and balance in healthy older adults.

**Methods:**

We systematically reviewed the publications in five databases. Studies that examine the effects of VRT on the measures of functional mobility and balance in healthy older adults were screened and included if eligible. Subgroup analyses were completed to explore the effects of different metrics of the intervention design (e.g., session time) on those outcomes related to functional mobility and balance.

**Results:**

Fifteen studies of 704 participants were included. The quality of these studies was good. Compared to traditional physical therapy (TPT), VRT induced greater improvement in TUG (MD = −0.31 s, 95% CI = −0.57 to −0.05, *p* = 0.02, *I*^2^ = 6.34%) and one-leg stance with open eyes (OLS-O) (MD = 7.28 s, 95% CI = 4.36 to 10.20, *p* = 0.00, *I*^2^ = 36.22%). Subgroup analyses revealed that immersive VRT with more than 800 min of total intervention time over 8 weeks and at least 120 min per week and/or designed by the two motor-learning principles was optimal for functional mobility and balance.

**Conclusion:**

Virtual reality training can significantly improve functional mobility and balance in healthy older adults compared to TPT, and the findings provided critical knowledge of the optimized design of VRT that can inform future studies with more rigorous designs.

**Systematic Review Registration:**

[https://www.crd.york.ac.uk/PROSPERO/], identifier [CRD42021297085].

## Introduction

Maintaining balance and functional mobility [i.e., the ability of people to move from one place to another and to participate in the activities of daily living (Forhan and Gill, 2013; Bouça-Machado et al., 2020)] is critical for older adults. Age and age-related diseases often diminish balance and functional mobility in older adults, which leads to decreased quality of life and increased risk of falling (Rubenstein, 2006; Liu and Latham, 2009; Lesinski et al., 2015). It is, thus, highly demanded to develop strategies to improve the balance and functional mobility for the older adult population.

Traditional physical training (TPT) that includes strength training and balance exercises has been shown beneficial for balance and functional mobility by primarily targeting musculoskeletal function (e.g., muscle strength) and the capacity to control posture when standing and walking *via* challenging the alignment of the body’s center of gravity concerning the base of support (Hrysomallis, 2011; Clemson et al., 2012; Lesinski et al., 2015). A recent study reported that, e.g., a 12-week balance training program could significantly improve the TUG and OLS performance of older women (FilipoviĆ et al., 2021). But it should be noted that the age-related decline of balance and functional mobility arises not only from the diminished musculoskeletal function but also from the structural and functional changes in visual and sensorimotor systems (de Amorim et al., 2018). A paradigm that aims at improving the functions in these multiple aspects may, thus, hold great potential to improve functional mobility and balance in older adults.

Virtual reality training (VRT) is a kind of exergame intervention, that is developed to simulate the real-life environment or the protocols of the training program in a totally virtual condition. It is different from augmented reality, which only enhances or augments, not simulates a real-time direct or indirect view of a physical real-world environment by adding virtual computer-generated information (Carmigniani et al., 2011). VRT combines the visual feedback control to provide an interactive and immersion experience to users (Chen et al., 2021). This technique enables the design of training protocols in a virtual environment and allows older adults to complete the tasks at home with much fewer constraints to the environment (Mao et al., 2014). VRT consists of appealing, motivating, and encouraging exercise concepts to different populations (Owens et al., 2011; Laufer et al., 2014) and can simultaneously strengthen muscles, facilitate brain activation, and improve sensory response, which leads to the increase of attention, motor control, and gait efficiency (Lee and Shin, 2013), which holds promise to serve as a novel rehabilitative strategy to restore and/or improve balance and functional mobility (Lee, 2020; Moreira et al., 2021).

However, significant variance in the design of study protocol (e.g., number of intervention sessions) and the characteristics of the VR system (e.g., immersive or not) were presented in the published study, which lead to the inconsistent observations in the effects of VRT on functional mobility and balance. Babadi and Daneshmandi (2021) observed that, e.g., a 9-week Xbox Kinect intervention significantly improved the time to complete the Timed Up and Go test (TUG), compared to TRT; but Stanmore et al. (2019) observed in another study that a 12-week Xbox Kinect intervention cannot induce greater improvement in TUG time in older adults who lived in nursing homes compared to TPT. Neri et al. (2017) completed a meta-analysis in 2017 and observed the benefits of VRT on balance and functional mobility in older adults as compared to the control (i.e., those who were inactive without doing PT). However, the risk of bias of this meta-analysis was relatively high, the number of the included studies was very limited (only six studies), and the observation of that study, thus, needs to be considered with caution. Additionally, since 2017, VR has been increasingly applied to older adults with the fast advances in VR technology (Phu et al., 2019) and many more studies have emerged. It is, thus, highly demanded to better characterize the efficacy of VRT on balance and functional mobility in older adults.

This study thus aims to quantitatively examine the effects of VRT on functional mobility and balance in healthy older adults compared to TRT by completing a systematic review and meta-analysis of the peer-reviewed publications. The results of this study will ultimately highlight recent research efforts for VRT, provide novel and critical knowledge into the effects of VRT on functional mobility and balance, and inform the rigorous design of future studies in this field.

## Methods

### Design

This systematic review and meta-analysis were conducted according to the Preferred Reporting Items for Systematic Reviews and Meta-Analyses (PRISMA) (Page et al., 2021). Review methods were registered prospectively on PROSPERO (CRD42021297085).

### Literature Search

The literature search was independently carried out by two researchers (ML and KZ). Searches for articles were conducted in four health-related, biomedical, and psychological databases (PubMed, Web of Science, SPORT-Discus, MEDLINE, and Science Direct). The literature search was driven from the inception of the publication until September 20, 2021. Functional mobility and balance are oftentimes assessed by the characteristics of gait, standing postural control, and transfers during the performance of a functional task; therefore, we used a comprehensive search strategy (Supplementary Table 1). Citation tracking of the articles and hand searching of important primary articles and review articles were also carried out.

### Selection Criteria

The studies were included in this review if they met the following criteria:

• Participants: (1) age ≥60 years; (2) intact cognitive function as assessed by neuropsychological tests or other objective measurements; (3) without overt neurological diseases (e.g., stroke, Parkinson’s disease, meningitis, etc.) that seriously affect sensory motor and autonomic dysfunction; (4) without visual impairment; (5) can stand and walk without personal assistance; (6) native to daily exercise (i.e., no habit of performing exercise daily).
• Interventions: VRT (e.g., VR games, VR, and fitness program).
• Comparison: TPT (e.g., balance training, and strength training).
• Outcomes: functional mobility test [e.g., TUG, functional reach test (FRT), one-leg stance (OLS), gait speed (GS), average sway speed, sway length, etc.].
• Study design: randomized controlled trials.
• Articles were excluded if (1) the language was non-English or unable to obtain outcome data; (2) review papers and conference articles.

### Data Extraction

The data extraction process was conducted according to the Cochrane Collaboration Handbook (Higgins et al., 2019). Two authors (KZ and ML) extracted the relevant data from the included studies in a standardized manner. The data were extracted as follows:

• The first author and publication time of the literature.
• Average age and sample size of the research subjects.
• Load, frequency, and period of interventions.
• Outcomes: The primary outcome is the TUG.
• The secondary outcomes are the FRT, One-leg stance with open eyes (OLS-O), and GS. Data format: mean ± SD.
• Key information for risk assessment of bias.

For each included study, the mean and the SD of post-test and follow-up tests were extracted. If any relevant data were missing, the corresponding author or authors were contacted by the first researcher *via* email.

### Quality Assessment

The quality of the included studies was assessed independently by two authors (ML and LZ) based on the guidance in the Cochrane Handbook for Systematic Reviews of Interventions (Higgins et al., 2019). The bias risk assessment mainly includes seven criteria: random sequence generation (selection bias), allocation concealment (selection bias), blinding of participants and personnel (performance bias), blinding of outcome assessment (detection bias), incomplete outcome data (attrition bias), selective reporting (reporting bias), and other bias. Any score on which the two authors disagreed was discussed with the third author (JZ or DB) until a consensus was achieved.

### Statistical Analysis

Revman 5.3 software (Cochrane Collaboration, Oxford, United Kingdom) and Stata version 16.0 (Stata Statistical Software, release 16; Stata Corp., College Station, TX, United States) were used for data synthesis and analysis. Continuous data were analyzed using the inverse variance approach by combining the mean difference (MD) of individual studies. The MD was calculated as the mean difference of the outcomes in the intervention group before and after the intervention minus the mean difference of the outcomes in the control group before and after the intervention (Cohen, 2013). The statistical heterogeneity was evaluated using heterogeneity chi-squared (χ^2^) and *I*^2^ values. The level of heterogeneity was interpreted according to the guidelines from Cochrane’s collaboration: *I*^2^ values of 25, 50, and 75% correspond to low, moderate, and high heterogeneity, respectively (Higgins et al., 2003). A random-effect model was used to conservatively estimate the pooled effect in anticipation of heterogeneity across individual studies due to differences in participant and intervention characteristics. In addition, publication bias was assessed by generating funnel plots and conducting Egger’s test. If significant asymmetry was detected (Egger’s test *p* < 0.1), we estimated the magnitude of the small study effect using the Trim and Fill method (Duval and Tweedie, 2000). Statistical significance was set at *p* < 0.05.

## Results

### Study Selection

**FIGURE 1:**
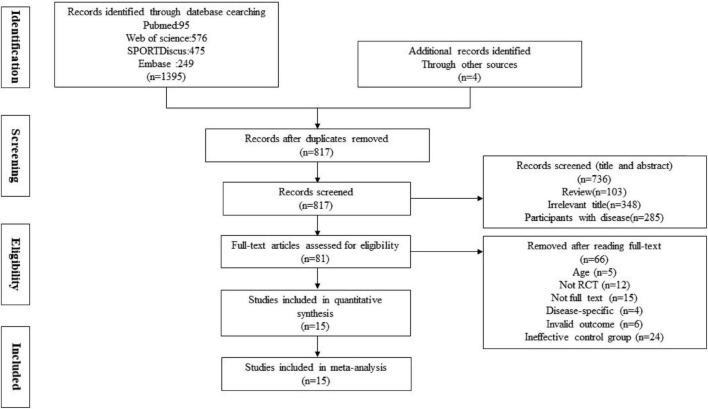
Preferred reporting items for systematic reviews and meta-analyses (PRISMA) flow chart of the study identification and selection process.

The flow of the study identification and selection process is summarized in Figure 1. The systematic search yielded 1,384 records: PubMed (*n* = 105), Web of Science (*n* = 570), SPORT-Discus (*n* = 56), MEDLINE (*n* = 428), EMBASE (*n* = 249), and Manual Search (*n* = 4). A total of 565 repetitive publications were excluded, leaving 819 articles. Then, 786 irrelevant articles were excluded by title and abstract, which consist of 33 publications with full text. Next, these 33 articles were further evaluated by reviewing the whole article, and 18 of them were excluded. Therefore, 15 publications were identified for inclusion in the systematic review and meta-analysis.

### Quality Assessment

**FIGURE 2:**
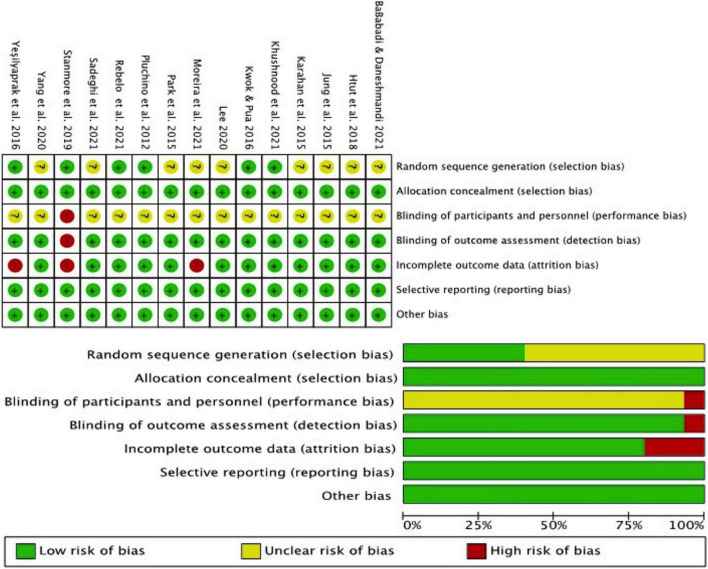
Risk of bias graph.

The result of the Cochrane risk of bias tool is summarized in Figure 2. The overall quality of the included articles was good. Most of the studies did not blind the participants, supervisors, or testing personnel. All the studies were at unclear or high risk of bias in at least one of the domains and were considered to be at possible risk of bias.

### Characteristics of Included Studies

#### Participants

The sample size of the intervention groups ranged from 8 to 49 subjects and a total of 704 (VRT group = 352, TPT group = 352). All participants were more than 60 years old (Table 1). The participants did not have previous experience with VRT and did not regularly exercise. One study only recruited female participants (Jung et al., 2015), one study only recruited male participants (Sadeghi et al., 2021), and other studies investigated both male and female participants. Six studies (Jung et al., 2015; Yeşilyaprak et al., 2016; Htut et al., 2018; Stanmore et al., 2019; Babadi and Daneshmandi, 2021; Rebelo et al., 2021) focused on participants in an institutionalized environment (hospital or nursing home); one study did not provide the related information (Khushnood et al., 2021), and other studies participants were recruited from the local community.

**TABLE 1:** Characteristics of participants in each study.

References	Country/location	Groups	Sample size (male/female)	Age (years) Mean ± *SD*	Population type
Pluchino et al., 2012	United States	TPT	8	76.0 ± 7.7	Community-dwelling
		VRT	8	70.7 ± 8.5	
Jung et al., 2015	South Korea	TPT	0/8	74.3 ± 3.5	Senior citizen center
		VRT	0/8	74.3 ± 2.1	
Park et al., 2015	South Korea	TPT	10/2	65.2 ± 7.9	Local community
		VRT	9/3	66.5 ± 8.1	
Karahan et al., 2015	South Korea	TPT	24/18	71.5 ± 4.7	Community-dwelling
		VRT	27/21	71.3 ± 6.1	
Yeşilyaprak et al., 2016	Turkey	TPT	2/9	73.1 ± 4.5	Nursing Home
		VRT	4/3	70.1 ± 4.0	
Kwok and Pua, 2016	Singapore	TPT	40	69.8 ± 7.5	Community-dwelling
		VRT	40	70.5 ± 6.7	
Htut et al., 2018	Myanmar	TPT	13/8	75.9 ± 5.7	Nursing Home
		VRT	12/9	75.8 ± 4.9	
Stanmore et al., 2019	United Kingdom	TPT	5/38	77.8 ± 10.2	Sheltered housing
		VRT	4/45	77.9 ± 8.9	
Lee, 2020	South Korea	TPT	28	79.4 ± 6.2	Local community
		VRT	28	81 ± 6.9	
Yang et al., 2020	Taiwan	TPT	1/9	68.7 ± 2.7	Local community
		VRT	1/9	67.5 ± 3.7	
Babadi and Daneshmandi, 2021	Iran	TPT	6/6	67.5 ± 3.1	Nursing Home
		VRT	6/6	66.5 ± 3.8	
Moreira et al., 2021	Brazil	TPT	34	70.8 ± 5.6	Local community
		VRT	32	70 ± 4.5	
Khushnood et al., 2021	Pakistan	TPT	25/16	60+	Unclear
		VRT	26/16	60+	
Rebelo et al., 2021	Brazil	TPT	2/15	71.4 ± 5.9	Local hospitals
		VRT	4/16	69.3 ± 5.7	
Sadeghi et al., 2021	Malaysia	TPT	14	70.4 ± 4.3	Local community
		VRT	15	74.1 ± 7	

*TPT, traditional physical training; VRT, virtual reality training.*

#### Intervention Characteristics

The information on the intervention parameters is included in Table 2. About the system implementing VRT, three studies focused on immersion VR systems (Yeşilyaprak et al., 2016; Lee, 2020; Rebelo et al., 2021); the VR systems used are Virtual Active, Oculus Rif, and BTS NIRVANA VR Interactive System, which can offer comprehensive content, immersive experience; other studies focused on not immersion VR including Nintendo Wii Fit (Pluchino et al., 2012; Jung et al., 2015; Park et al., 2015; Kwok and Pua, 2016; Khushnood et al., 2021) and Xbox Kinect (Karahan et al., 2015; Htut et al., 2018; Stanmore et al., 2019; Yang et al., 2020; Babadi and Daneshmandi, 2021; Moreira et al., 2021; Sadeghi et al., 2021), which can only provide feedback through a smaller screen and cannot provide an immersive experience.

**TABLE 2:** Characteristics of the interventional protocol.

References	Groups	Interventions	Design principles	Duration (min/session)	Frequency (days/week)	Period (weeks)	Outcome
Pluchino et al., 2012	TPT	Balance training, strength training	The task difficulty is progressively increased according to the user’s ability	60	2	8	TUG→ FRT→ OLS-O→
	VRT	Wii, balance board (soccer heading, skislalom, skijump, tabletilt, tightrope, riverbubble, penguinslide, and lotusfocus)		60	2	8	
Jung et al., 2015	TPT	Balance training, strength training	Repetitive, varied practice of meaningful tasks	30	2	8	FRT↓ TUG↑ GS→
	VRT	Wii: wakeboard, frisbeedog, jet ski, canoegame		30	2	8	
Park et al., 2015	TPT	Balance training, strength training	Repetitive, varied practice of meaningful tasks	30	3	8	TUG↑
	VRT	Wii balance board: soccer heading, snowboard slalom, tabletilt		30	3	8	
Karahan et al., 2015	TPT	Balance training, strength training, flexibility	Repetitive, varied practice of meaningful tasks	30	5	6	TUG↑
	VRT	VRT: “Kinect Adventures, Kinect Sports, and Kinect Sports Season two”		30	5	6	
Yeşilyaprak et al., 2016	TPT	Balance training	Sensory feedback that is related to the task is necessary	35–45	3	6	TUG→ OLS-O→ OLS-C→
	VRT	VR-based balance exercises		35–45	3	6	
Kwok and Pua, 2016	TPT	Balance training	Repetitive, varied practice of meaningful tasks	60	1	12	TUG→ GS→
	VRT	Wii balance board and resistance band		60	1	12	
Htut et al., 2018	TPT	Balance training, strength training	The task difficulty is progressively increased according to the user’s ability	30	3	8	TUG→ 5TSTS↓
	VRT	X-box 360: Light Raise, Virtual Smash, Stack’em Up, One Ball Roll, Pin Push, Super Saver, Target Kick, Play Paddle Panic, Body Bally, Bamp Bash.		30	3	8	
Stanmore et al., 2019	TPT	OTAGO strength and balance home exercise program	The task difficulty is progressively increased according to the user’s ability	30	3	12	TUG→
	VRT	Kinect: lower or upper limb exercises			3	12	
Lee, 2020	TPT	Gait training	Sensory feedback that is related to the task is necessary	50	5	4	OLS-O↑ FRT→ TUG↑ GS→
	VRT	Gait training with virtual reality		50	5	4	
Yang et al., 2020	TPT	Balance training	Repetitive, varied practice of meaningful tasks	45	2	5	TUG→ FRT↑ OLS-O→ OLS-C→ 30s-CST→
	VRT	Xbox: Zen Energy, Yoga, Destination Bollywood, Cardio Boxing, Humana Vatality and Cardio		45	2	5	
Babadi and Daneshmandi, 2021	TPT	Balance training	Repetitive, varied practice of meaningful tasks	60	3	9	TUG→ FRT→ OLS-O→
	VRT	Xbox: boxing, table tennis, soccer, golf, skis, and American football.		60	3	9	
Moreira et al., 2021	TPT	Strength training	Repetitive, varied practice of meaningful tasks	50	3	12	TUG→ GS↑ 5TSTS→
	VRT	Xbox: strength exercise, squats and lunges. balance and cardiorespiratory exercises consisted of boxing and lateral and anteroposterior displacements		50	3	12	
Khushnood et al., 2021	TPT	Balance training	Repetitive, varied practice of meaningful tasks	30	2	8	TUG→
	VRT	Wii Fit: single-leg stand up, tandem walking, tiptoe walk, walk on heels, sideways walk, walking with high steps and contra-lateral arm raise		30	2	8	
Rebelo et al., 2021	TPT	Balance training	Sensory feedback that is related to the task is necessary	50	2	8	TUG→ FRT→
	VRT	Oculus Rif: BoxVR, Baskhead, InCell, and Thrills and Chills Roller Coasters		50	2	8	
Sadeghi et al., 2021	TPT	Balance training	The task difficulty is progressively increased according to the user’s ability	40	3	8	TUG→ OLS-O↑ GS→
	VRT	Xbox: Light Race (Stomp It), Target Kick, Goalkeeper		40	3	8	

*TPT, traditional physical training; VRT, virtual reality training; TUG, Timed Up and Go; OLS-O, one-leg stance with open eyes; FRT, functional reach test; GS, gait speed; 30s-CST, 30 s chair stand test; 5TSTS, 5-time sit-to-stand test; →, no significant difference between groups; ↑, VRT group was significantly better than PT group; ↓, PT group was significantly better than VRT group.*

The different game was designed based on the different motor-learning principles (Levin et al., 2015); we refer to the previous meta to divide the game type (Chen et al., 2021). In our study, the VR interventions can be categorized into three types (Table 2). Eight of the included studies implemented interventions designed by the principle of *Repetitive, varied practice of meaningful tasks* (Jung et al., 2015; Karahan et al., 2015; Park et al., 2015; Kwok and Pua, 2016; Yang et al., 2020; Babadi and Daneshmandi, 2021; Khushnood et al., 2021; Moreira et al., 2021), four studies designed by the principle of *The task difficulty is progressively increased according to the user’s ability* (Pluchino et al., 2012; Htut et al., 2018; Stanmore et al., 2019; Sadeghi et al., 2021), and other three studies are based on the combination of the two principles *Repetitive, varied practice of meaningful tasks* and *Sensory feedback that is related to the task is necessary* (Yeşilyaprak et al., 2016; Lee, 2020; Rebelo et al., 2021). The intervention duration was from 3 to 15 weeks, with a frequency between one and five times per week. The session time for most group classes varied from 30 to 50 min (Table 2).

In terms of TPT of the control group, seven studies only used balance training (Kwok and Pua, 2016; Yeşilyaprak et al., 2016; Babadi and Daneshmandi, 2021; Khushnood et al., 2021; Rebelo et al., 2021; Sadeghi et al., 2021); six studies used balance combined with strength training (Pluchino et al., 2012; Jung et al., 2015; Karahan et al., 2015; Park et al., 2015; Htut et al., 2018; Stanmore et al., 2019); one study used gait training (Lee, 2020); and one study used strength training (Moreira et al., 2021) (Table 2).

### Study Outcomes

Outcomes for assessing functional mobility included the following (Table 2): TUG (Pluchino et al., 2012; Jung et al., 2015; Karahan et al., 2015; Park et al., 2015; Kwok and Pua, 2016; Yeşilyaprak et al., 2016; Htut et al., 2018; Stanmore et al., 2019; Lee, 2020; Yang et al., 2020; Babadi and Daneshmandi, 2021; Khushnood et al., 2021; Moreira et al., 2021; Rebelo et al., 2021; Sadeghi et al., 2021), GS (Jung et al., 2015; Kwok and Pua, 2016; Lee, 2020; Moreira et al., 2021; Sadeghi et al., 2021), OLS-O (Pluchino et al., 2012; Yeşilyaprak et al., 2016; Lee, 2020; Yang et al., 2020; Sadeghi et al., 2021), FRT (Pluchino et al., 2012; Jung et al., 2015; Lee, 2020; Yang et al., 2020; Babadi and Daneshmandi, 2021; Rebelo et al., 2021), OLS-C (Yeşilyaprak et al., 2016; Yang et al., 2020), 30-s chair stand test (30s-CST) (Yang et al., 2020), and 5-time sit-to-stand test (5TSTS) (Htut et al., 2018; Moreira et al., 2021).

### Effects of Virtual Reality Training on Functional Mobility and Balance

Fifteen studies reported intervention effects on functional mobility using TUG tests (Pluchino et al., 2012; Jung et al., 2015; Karahan et al., 2015; Park et al., 2015; Kwok and Pua, 2016; Yeşilyaprak et al., 2016; Htut et al., 2018; Stanmore et al., 2019; Lee, 2020; Yang et al., 2020; Babadi and Daneshmandi, 2021; Khushnood et al., 2021; Moreira et al., 2021; Rebelo et al., 2021; Sadeghi et al., 2021), but inconsistent results were observed when comparing the effects between VRT and TPT. Four studies (Jung et al., 2015; Karahan et al., 2015; Park et al., 2015; Lee, 2020) showed that VRT could improve participants’ TUG compared to TPT. Nevertheless, the other eleven studies found that VRT could not improve participants’ TUG compared to TPT (Pluchino et al., 2012; Kwok and Pua, 2016; Yeşilyaprak et al., 2016; Htut et al., 2018; Stanmore et al., 2019; Yang et al., 2020; Babadi and Daneshmandi, 2021; Khushnood et al., 2021; Moreira et al., 2021; Rebelo et al., 2021; Sadeghi et al., 2021).

Six studies reported intervention effects on FRT distance, four of the included studies observed no significant difference between VRT and TPT (Pluchino et al., 2012; Lee, 2020; Babadi and Daneshmandi, 2021; Rebelo et al., 2021), one study observed that VRT significantly improved FRT as compared to TPT (Yang et al., 2020), and another one study observed that TPT significantly improved FRT as compared to VRT (Jung et al., 2015).

Six studies reported results for OLS-O performance (Pluchino et al., 2012; Yeşilyaprak et al., 2016; Lee, 2020; Yang et al., 2020; Babadi and Daneshmandi, 2021; Sadeghi et al., 2021), four of the included studies observed no significant difference between VRT and TPT (Pluchino et al., 2012; Yeşilyaprak et al., 2016; Yang et al., 2020; Babadi and Daneshmandi, 2021), and other two studies observed that VRT significantly improved OLS-O as compared to TPT (Lee, 2020; Sadeghi et al., 2021). There was no significant difference between VRT and TPT that two studies reported OLS-C performance (Yeşilyaprak et al., 2016; Yang et al., 2020).

Five studies examined the effects of VRT on GS (Jung et al., 2015; Kwok and Pua, 2016; Lee, 2020; Moreira et al., 2021; Sadeghi et al., 2021), only one study observed that VRT significantly improved FRT as compared to TPT (Moreira et al., 2021), other four studies observed no significant difference between VRT and TPT (Jung et al., 2015; Kwok and Pua, 2016; Lee, 2020; Sadeghi et al., 2021).

One study reported results for 30s-CST performance (Yang et al., 2020), two studies reported results for 5TSTS performance (Htut et al., 2018; Moreira et al., 2021), only one study observed that TPT significantly improved 5TSTS as compared to VRT (Htut et al., 2018), and other studies observed no significant difference between VRT and TPT (Moreira et al., 2021).

### Meta-Analysis

Based upon the information from the review, we performed the subgroup analyses to compare the effects between VRT types (i.e., immersive or not), intervention length (i.e., length of <8 weeks or length of ≥8 weeks), frequency (<3 or ≥3 times/week), total intervention time (<800 or ≥800 min), and different VR game types categorized following the motor-learning principles (Levin et al., 2015; Yang et al., 2020).

**FIGURE 3:**
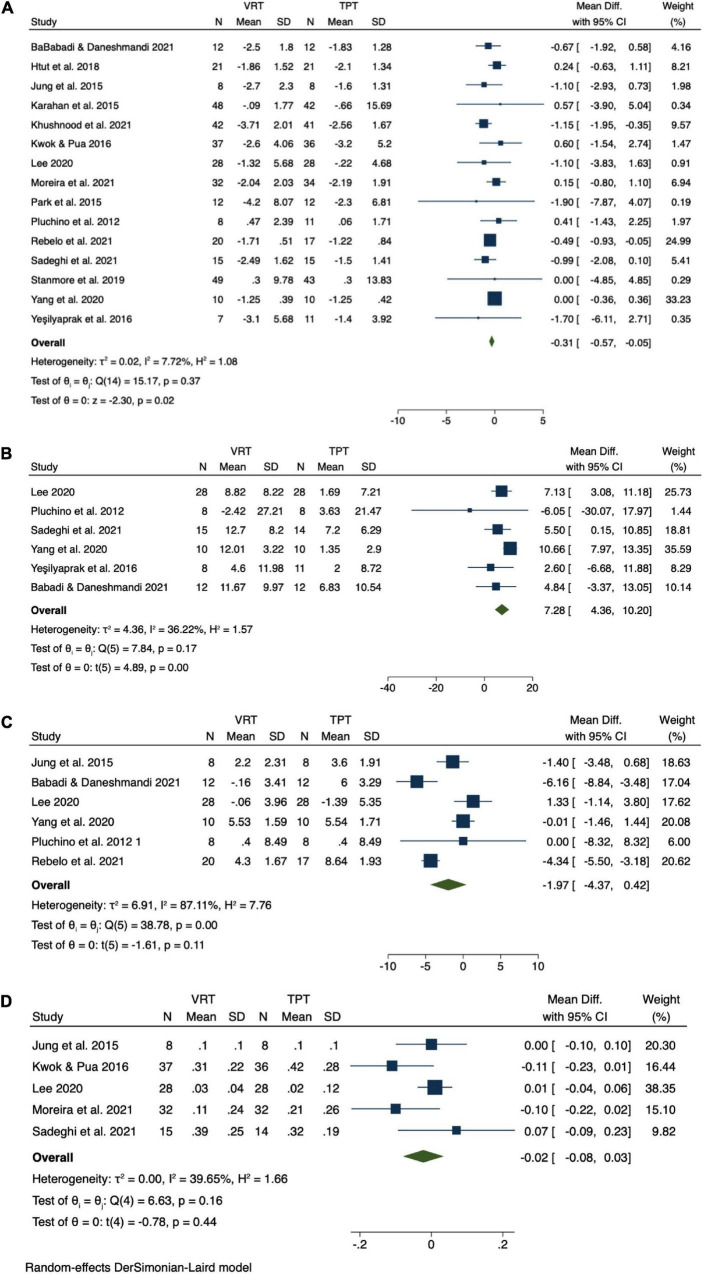
Result of meta-analysis. **(A)** Timed Up and Go (TUG), **(B)** one-leg stance with open eyes (OLS-O), **(C)** functional reach test (FRT), and **(D)** gait speed (GS).

**FIGURE 4:**
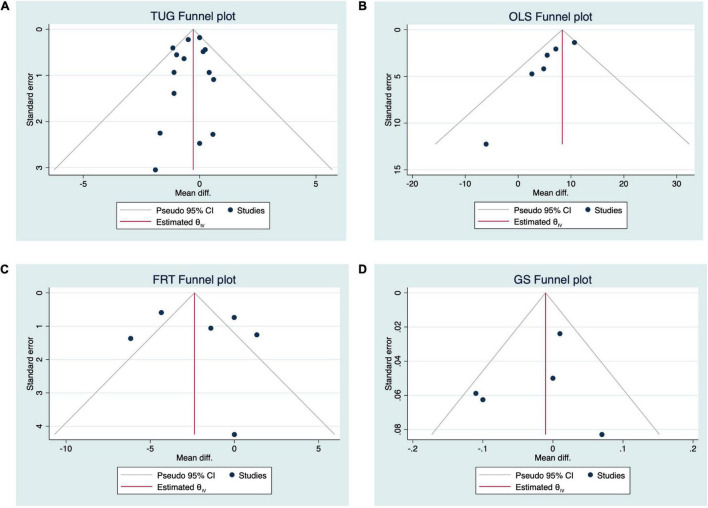
Funnel plots.

The results of meta-analysis demonstrated compared to TPT, VRT induced significant improvement in TUG. The pooled effect size was significant (MD = −0.31 s, 95% CI = −0.57 to −0.05, *p* = 0.02, Figure 3A) and with low heterogeneity (*I*^2^ = 6.34%, *p* = 0.37). The funnel plot (Figure 4A) and Egger’s test (*t* = −0.60, *p* = 0.557) indicated the evidence of symmetry. The subgroup analysis demonstrated (1) the immersive VRT induced greater improvement (MD = −0.52 s, 95% CI = −0.95 to −0.05, *p* = 0.02) than not immersive; (2) the longer sessions’ length (≥8 weeks, MD = −0.45 s, 95% CI = −0.77 to −0.13, *p* = 0.01, *I*^2^ = 5.17%) induced greater improvement than shorter sessions length (<8 weeks); (3) the intervention with time longer than 120 min per week (MD = −0.42 s, 95% CI = −0.79 to −0.05, *p* = 0.03, *I*^2^ = 0%) helped more compared to those with time less than 120 min per week; (4) the total intervention time of at least 800 min (MD = −0.38 s, 95% CI = −0.75 to −0.01, *p* = 0.04, *I*^2^ = 0%) demonstrated a significant greater effect size than those with time less than 800 min; and (5) the combination of the two principles *Repetitive, varied practice of meaningful tasks* and *Sensory feedback that is related to the task is necessary* (MD = −0.52 s, 95% CI = −0.95 to 0.08, *p* = 0.02, *I*^2^ = 0%) was the strategy with greatest effects on balance and functional mobility (Table 3).

**TABLE 3:** Overall and subgroup analysis results regarding the effects of VRT on balance and functional mobility.

Outcomes	Overall and Subgroup analysis	Number of studies	WMD (95% CI)	*P*-value	Test of heterogeneity		
					χ*^2^*	*P*-value	*I*^2^(%)
TUG	Overall	15	−0.31 (−0.57, −0.05)	0.02	15.17	0.37	7.72
	Immersion						
	Yes	3	−0.52 (−0.95, 0.08)	0.02	0.47	0.79	0
	No	12	−0.26 (−0.61, 0.09)	0.14	13.08	0.29	15.92
	Frequency (times/week)						
	<3	6	−0.38 (−0.85, 0.08)	0.11	9.81	0.08	49.02
	≥3	9	−0.25 (−0.73, 0.24)	0.32	5.34	0.72	0
	Length (weeks)						
	<8	4	−0.03 (−0.38, 0.32)	0.89	1.24	0.74	0
	≥8	11	−0.45 (−0.77, −0.13)	0.01	10.54	0.39	5.17
	Weekly intervention time						
	<120	9	−0.33 (−0.80, 0.14)	0.17	11.91	0.16	32.84
	≥120	6	−0.42 (−0.79, −0.05)	0.03	2.38	0.79	0
	Total intervention time (min)						
	<800	9	−0.39 (−0.89, 0.10)	0.12	11.92	0.15	32.89
	≥800	6	−0.38 (−0.75, −0.01)	0.04	2.79	0.73	0
	Design principles						
	*Repetitive, varied practice of meaningful tasks*	8	−0.33 (−0.80, 0.14)	0.17	9.64	0.21	27.39
	*The task difficulty is progressively increased according to the user’s ability*	4	−0.17 (−0.88, 0.55)	0.64	3.43	0.33	12.60
	*Repetitive, varied practice of meaningful tasks + Sensory feedback that is related to the task is necessary*	3	−0.52 (−0.95, 0.08)	0.02	0.47	0.79	0
OLS-O	Overall	6	7.28 (4.36, 10.20)	0.00	7.84	0.17	36.22
	Immersion						
	Yes	2	6.41 (2.69, 10.12)	0.00	0.77	0.38	0
	No	4	7.48 (3.16, 11.79)	0.00	5.60	0.13	46.43
	Frequency (times/week)						
	<3	2	6.74 (−7.13, 20.62)	0.34	1.84	0.18	45.55
	≥3	4	5.96 (3.10, 8.82)	0.00	0.92	0.82	0
	Length (weeks)						
	<8	3	8.30 (4.59, 12.01)	0.00	4.08	0.13	51.00
	≥8	3	4.92 (0.52, 9.33)	0.03	0.85	0.65	0
	Weekly intervention time						
	<120	3	1.25 (−0.53, 3.03)	0.17	18.59	0.00	89.24
	≥120	3	6.14 (2.76, 9.52)	0.00	0.88	0.64	0
	Total intervention time (min)						
	<800	3	7.88 (2.62, 13.15)	0.00	4.47	0.11	55.31
	≥800	4	6.40 (2.81, 9.99)	0.00	1.30	0.52	0
	Design principles						
	*Repetitive, varied practice of meaningful tasks*	2	9.10 (4.04, 14.15)	0.00	1.74	0.19	42.69
	*The task difficulty is progressively increased according to the user’s ability*	2	4.95 (−0.27, 10.17)	0.06	0.85	0.36	0
	*Repetitive, varied practice of meaningful tasks + Sensory feedback that is related to the task is necessary*	2	6.41 (2.69, 10.12)	0.00	0.77	0.38	0
FRT	Overall	6	−1.97 (−4.37, 0.42)	0.11	38.78	0.00	87.11
	Immersion						
	Yes	2	−1.61 (−7.17, 3.94)	0.57	16.64	0.00	93.99
	No	4	−2.13 (−4.37, 0.42)	0.16	15.76	0.00	80.96
	Frequency (times/week)						
	<3	4	−1.80 (−4.52, 0.92)	0.19	22.43	0.00	86.63
	≥3	2	−2.40 (−9.74, 4.94)	0.52	16.25	0.00	93.84
	Length (weeks)						
	<8	2	0.33 (−0.91, 1.58)	0.60	0.31	0.36	0
	≥8	4	−3.65 (−5.86, −1.44)	0.00	9.69	0.00	69.05
	Weekly intervention time						
	<120	3	0.46 (−1.63, 0.72)	0.45	1.17	0.56	0
	≥120	3	−3.08 (−6.89, 0.72)	0.11	20.39	0.00	90.19
	Total intervention time (min)						
	<800	2	−0.50 (−1.80, 0.80)	0.45	1.16	0.34	13.66
	≥800	4	−2.73 (−6.22, 0.76)	0.13	21.14	0.00	85.81
	Design principles						
	*Repetitive, varied practice of meaningful tasks*	3	−2.36 (−5.63, 0.91)	0.16	15.65	0.00	87.22
	*The task difficulty is progressively increased according to the user’s ability*	1	0.00 (−8.32, 8.32)	1.0	–	–	–
	*Repetitive, varied practice of meaningful tasks + Sensory feedback that is related to the task is necessary*	2	−1.61 (−7.17, 3.94)	0.57	16.64	0.00	93.99
GS	Overall	5	−0.02 (−0.08, 0.03)	0.44	6.63	0.16	39.65
	Immersion						
	Yes	1	0.01 (−0.04, 0.06)	0.68	0	–	–
	No	4	−0.04 (−0.12, 0.03)	0.28	4.72	0.19	36.49
	Frequency (times/week)						
	<3	2	0.05 (−0.16, 0.06)	0.36	2.03	0.15	50.72
	≥3	3	−0.01 (−0.08, 0.07)	0.84	3.44	0.18	41.89
	Length (weeks)						
	<8	1	0.01 (−0.04, 0.06)	0.68	0	–	–
	≥8	4	−0.04 (−0.12, 0.03)	0.28	4.72	0.19	36.49
	Weekly intervention time						
	<120	3	−0.02 (−0.12, 0.07)	0.64	3.65	0.16	45.18
	≥120	2	−0.03 (−0.13, 0.07)	0.57	2.70	0.10	62.94
	Total intervention time (min)						
	<800	3	−0.02 (−0.12, 0.07)	0.64	3.65	0.16	45.18
	≥800	2	−0.03 (−0.13, 0.07)	0.57	2.70	0.10	62.94
	Design principles						
	*Repetitive, varied practice of meaningful tasks*	3	−0.06 (−0.14, 0.01)	0.09	2.57	0.28	0
	*The task difficulty is progressively increased according to the user’s ability*	1	0.07 (−0.09, 0.23)	0.40	0	–	–
	*Repetitive, varied practice of meaningful tasks + Sensory feedback that is related to the task is necessary*	1	0.01 (−0.04, 0.06)	0.68	0	–	–

*TUG, Timed Up and Go; OLS-O, one-leg stance with open eyes; FRT, functional reach test; GS, gait speed.*

Specifically, for OLS-O, compared to TPT, VRT induced significant improvement in it. The pooled effect size was significant (MD = 7.28 s, 95% CI = 4.36 to 10.20, *p* = 0.00, Figure 3B) and with moderate heterogeneity (*I*^2^ = 36.22%, *p* = 0.37). The funnel plot (Figure 4B) and Egger’s test (Egger’s test, *t* = −2.54, *p* = 0.064) indicated that there may be publication bias on these results, but the Trim and Fill method for sensitive analysis showed that the pooled ES was robust. The subgroup analysis showed that interventions with both immersive (MD = 6.41 s, 95% CI = 2.69 to 10.12, *p* = 0.00) or not immersive design (MD = 7.48 s, 95% CI = 3.16 to 11.79, *p* = 0.00, moderate heterogeneity: *I*^2^ = 46.43%, *p* = 0.13), high frequency (≥3 times/week, MD = 5.96 s, 95% CI = 3.10 to 8.82, *p* = 0.00), weekly intervention time than 120 min (MD = 6.14 s, 95% CI = 2.76 to 9.52, *p* = 0.00, *I*^2^ = 0%), both shorter sessions’ length (<8 weeks, MD = 7.88 s, 95% CI = 2.62 to 13.15, *p* = 0.00, *I*^2^ = 55.31%) and longer sessions’ length (≥8 weeks, MD = 6.40 s, 95% CI = 2.81 to 9.99, *p* = 0.00, *I*^2^ = 0%), or longer sessions’ length (≥8 weeks, MD = −0.45 s, 95% CI = −0.77 to −0.13, *p* = 0.01, *I*^2^ = 5.17%), or based on the combination of the two principles *Repetitive, varied practice of meaningful tasks* and *Sensory feedback that is related to the task is necessary* or upon *the principle of Repetitive, varied practice of meaningful tasks* (MD = 9.10 s, 95% CI = 4.04 to 14.15, *p* = 0.00, *I*^2^ = 42.69%), can induce significant improvements in OLS-O (Table 3).

For FRT and GS, the meta-analysis demonstrated that compared to TPT, VRT did not induce significantly greater improvement in either FRT (MD = −1.97 cm, 95% CI = −4.37 to 0.42, *p* = 0.11, *I*^2^ = 87.11%, Figure 3C) or GS (MD = −0.02 m/s, 95% CI = −0.08 to 0.03, *p* = 0.44, *I^2^* = 39.65%, Figure 3D). The funnel plots (Figures 4C,D) (FRT: *t* = 0.36, *p* = 0.735; GS: *t* = −0.37, *p* = 0.738) are symmetrical. Significant discrepancies were not observed in our analyses of other subgroups (Table 3). Due to the lack of enough number of research on 30s-CST and 5TSTS, we did not perform the analysis on these two outcomes.

## Discussion

Our systematic review and meta-analysis suggest that VRT is a promising strategy to improve functional mobility and balance in healthy older adults. All the studies included here were of good quality. Specifically, compared to TRT, the VRT induced significantly greater improvements in the performance of TUG and OLS-O; and subgroup analyses reveal that the immersive type of VR consisting of longer intervention sessions (≥8 weeks) and more time per week (≥800 min), with high frequency (≥3 times/week), and/or based on the principle of *“combined repetitive, varied practice of meaningful tasks* with *Sensory feedback that is related to the task is necessary”* can induce significant benefits for the functional mobility and balance in older adults. These elements of VRT would be appropriate and optimal in future studies and can be taken into consideration in future rehabilitative clinics.

It is observed here that the VRT induced significantly greater improvements in the performance of TUG and OLS-O as compared to TPT. The findings of this study are consistent with the previous meta-analysis study (Neri et al., 2017). However, we here provide novel evidence that even compared TRT [only inactive control was compared previously (Neri et al., 2017)], VRT can significantly enhance functional mobility and balance in older adults. VRT simulates the reality of the environment with interactive visual feedback technology, which is different from TPT. The multi-type feedback in the VR system may have a unique and significant impact on the intervention effect on balance and functional mobility. VRT can also benefit cognitive function and mood, another two important elements for the regulation of balance and functional mobility. Additionally, VRT is more enjoyable and attractive (Hall et al., 2016), in which participants do more interesting and hobby-like practices that can potentially increase the motivation of the participants to the exercise or training of VRT (McEwen et al., 2014; Whyatt et al., 2015; Padala et al., 2017; Buckinx et al., 2020). Taken together, the benefits of VRT on balance and functional mobility arise from the simultaneous enhancement of those multiple aspects pertaining to balance and functional mobility as induced by VRT.

The outcomes we measured in this study (e.g., TUG time) have been widely used to assess functional mobility and balance; both less TUG time and longer duration of OLS time suggest better balance and mobility. These outcomes have also been used to estimate the risk of risk events (e.g., falls) in older adults (Yeşilyaprak et al., 2016). For example, Barry et al. (2014) observed that older adults with a TUG time greater than 11–13.5 s had a significantly increased risk of falls. Here, we also observed a significant increase in them as induced by VRT (e.g., the difference in TUG time was 0.31 s), which indicates VRT may bring clinically meaningful improvements in functional mobility and balance. Future studies are warranted to explore to what extent the VRT-induced functional improvements can help the prevention of falls in older adults.

The subgroup analysis revealed that the immersive VRT induced greater improvement in functional mobility. Immersive VRT enables more visual feedback through a larger screen and accurate motion capture. Thus, learning through immersive VRT facilitates observation action and visual-spatial networks (van Diest et al., 2014). Our results also suggest that sessions with a duration of over 8 weeks could induce greater improvements than fewer than 8 weeks; the higher the weekly frequency and time of intervention, the large effects. This is consistent with the previous evidence which shows the effects of exergame (one type of VRT) on balance in older adults (Chen et al., 2021). Furthermore, the VRT designed based upon the combination of the two principles is more appropriate.

It should be noted that the effects of *VRT* on the performance of FRT and GS were not superior to TPT. Recent studies have shown that the TPT can induce comparable improvements in the proprioceptive and vestibular systems as VRT (Babadi and Daneshmandi, 2021) and a greater increase in muscle endurance or strength than VRT. Therefore, it would be reasonable that VRT had comparable but not significantly different effects in the performance of GS and FRT as compared to TPT.

As a recent popular technology-assisted exercise, VR-based balance exercises can be a convenient option because they can be more exciting while relieving the monotony of conventional exercises. However, the subjective willingness of using it, especially in older adults, influences much on the implementation of VRT-based intervention, especially when it is used by older adults themselves. Therefore, future efforts in this field would put more on improving the degree of satisfaction of users, lower the costs to make it more affordable to more populations (Mao et al., 2014), which will ultimately boost the implementation of VRT-based intervention.

Several limitations need to be noted. The number of included studies is still relatively small, which limits the scope of the analysis. Due to the limited number of studies and the selection of game types, the effects of different VR systems or game types on the outcomes were not examined. Only effects of VRT on relatively healthy older adults were examined, and it is highly demanded to examine whether VRT is beneficial to other populations (e.g., those with cognitive impairment). In addition, the experience or knowledge of using VRT is one important factor that contributes to the effects of VRT, which, however, did not explicitly assess in the previous studies; compared to TRT, VRT is implemented in a relatively new environment, which may potentially influence the effects of VRT. Future studies should carefully assess and include the subjective feeling on VRT of older adults and the environment when using it and include these aspects into the analysis. Due to the limited number of available outcomes used in those previous publications, the outcomes we chose in this review may not be the most appropriate. Moreover, the literature included in this study lacked long-term follow-up assessment. Future studies with longer-term follow-ups are, thus, needed to characterize how long the effects induced by VRT can sustain, which will be helpful for the design of rehabilitative programs using VRT. Additionally, the studies with positive results are more likely to be published, so there may be some publication bias. More studies that examine the effects of VRT are, thus, highly demanded. The heterogeneity in the included studies is still relatively high. The results, thus, need more caution even though the sensitivity analysis showed robust results.

## Conclusion

Nevertheless, to our knowledge, this is the first systematic review and meta-analysis that explicitly examines the effects of VRT on the functional mobility of healthy older adults based upon only RCTs with high quality. The knowledge provided by our study will ultimately inform the design of future studies implementing VRT with rigorous designs to examine the effects of VRT on functional mobility and balance in older adults and confirm the findings and conclusions in our study.

## Data Availability Statement

The original contributions presented in the study are included in the article/Supplementary Material, further inquiries can be directed to the corresponding author.

## Author Contributions

KZ, DB, ML, and JZ: design and/or conceptualization of the study. KZ, ML, YC, LZ, JZ, and DB: analysis and/or interpretation of the data. KZ, JZ, and DB: drafting and/or revising the manuscript. All authors contributed to the article and approved the submitted version.

## Conflict of Interest

The authors declare that the research was conducted in the absence of any commercial or financial relationships that could be construed as a potential conflict of interest.

## Publisher’s Note

All claims expressed in this article are solely those of the authors and do not necessarily represent those of their affiliated organizations, or those of the publisher, the editors and the reviewers. Any product that may be evaluated in this article, or claim that may be made by its manufacturer, is not guaranteed or endorsed by the publisher.
