# Supplementary material for: Is Virtual Reality Training More Effective Than Traditional Physical Training on Balance and Functional Mobility in Healthy Older Adults? A Systematic Review and Meta-Analysis
Source: Front Hum Neurosci. 2022 Mar 23;16:843481. doi: 10.3389/fnhum.2022.843481 (PMC8984187; doi:10.3389/fnhum.2022.843481)
Supplement: Supplementary file 1 [file Table_1.DOCX]

Supplementary Material

# Supplementary Table

**Supplementary Table S1. Search Strategy**

| Process | Keywords |
| --- | --- |
| # 1 | Virtua* Reality* OR VR OR Exergame* OR Exer-game* OR Video-game* OR Video-based OR Computer-based OR Wii OR Nintendo OR X-box OR Kinect |
| # 2 | Functional mobility OR Postur* Control OR Fall* OR Gait OR Walk* OR Mobility OR chair stand |
| # 3 | Elder* OR Aged OR Old* OR Senior* |
| # 4 | Randomized controlled trial OR Randomized OR RCT |
| # 5 | #1 AND #2 AND #3 AND #4 |
